# Superior Diagnostic Yield of EBUS-Guided Cryobiopsy over Needle Aspiration in Isolated, Mass-Negative Mediastinal Lymphadenopathy: A Prospective Within-Patient Study

**DOI:** 10.3390/diagnostics16111713

**Published:** 2026-06-02

**Authors:** Nilüfer Yiğit, Erhan Uğurlu, Meryem Sanlıalp, Emel Kılıçarslan, Ilknur Hatice Akbudak, Hande Senol

**Affiliations:** 1 Department of Pulmonology, Pamukkale University, 20160 Denizli, Türkiye; drerhanugurlu@gmail.com (E.U.); sanlialpmeryem@gmail.com (M.S.); 2 Department of Pathology, Pamukkale University, 20160 Denizli, Türkiye; emelkarpuzoglu@gmail.com; 3 Department of Anesthesia and Resuscitation, Pamukkale University, 20160 Denizli, Türkiye; ihaticea@pau.edu.tr; 4 Department of Biostatistics, Pamukkale University, 20160 Denizli, Türkiye; handesenol@gmail.com

**Keywords:** endobronchial ultrasound-guided transbronchial needle aspiration, EBUS-guided transbronchial mediastinal cryobiopsy, mediastinal lymphadenopathy, diagnostic yield, lymph node biopsy

## Abstract

**Background:** Endobronchial ultrasound-guided transbronchial needle aspiration (EBUS-TBNA) is the standard minimally invasive technique for mediastinal lymph node sampling; however, its diagnostic performance is limited in diseases requiring preserved tissue architecture, such as lymphoma and granulomatous disorders. EBUS-guided transbronchial mediastinal cryobiopsy (EBUS-TBMC) has emerged as a technique capable of obtaining larger, histologically intact samples. Evidence comparing these modalities in isolated mediastinal lymphadenopathy is limited. **Methods:** In this prospective, single-center, head-to-head cohort study, 89 consecutive patients with isolated mediastinal lymphadenopathy without parenchymal lung masses were enrolled. All patients underwent sequential EBUS-TBNA followed by EBUS-TBMC within the same session. The primary outcome was diagnostic yield; secondary outcomes included tissue adequacy and procedure-related complications. **Results:** EBUS-TBMC demonstrated a significantly higher diagnostic yield than EBUS-TBNA (83.1% vs. 28.0%, *p* < 0.001). TBMC established a diagnosis in 74/89 patients, whereas TBNA was diagnostic in 25/89. TBMC provided an additional diagnosis in 49 patients with non-diagnostic TBNA, while no case was diagnosed exclusively by TBNA. Superiority was consistent across malignant and benign conditions, particularly lymphoma and granulomatous diseases. Diagnostic yield was higher when ≥3 cryobiopsy samples were obtained (86.6% vs. 42.8%). Bleeding events were more frequent with TBMC but were mild-to-moderate, managed bronchoscopically, and no major complications or life-threatening events occurred. **Conclusions:** In isolated, mass-negative mediastinal lymphadenopathy, EBUS-TBMC provides a substantially higher diagnostic yield than TBNA. Early integration of TBMC may improve diagnostic efficiency and reduce the need for repeat or surgical procedures.

## 1. Introduction

Endobronchial ultrasound (EBUS)-guided transbronchial needle aspiration (TBNA) is currently the preferred minimally invasive technique for the diagnostic evaluation and mediastinal staging of hilar and mediastinal lymph nodes detected by computed tomography (CT) or positron emission tomography (PET), particularly in patients with suspected or confirmed lung cancer [1,2,3]. Owing to its high diagnostic yield and favorable safety profile, EBUS-TBNA has become the standard first-line approach for mediastinal lymph node sampling.

Despite its excellent performance in primary pulmonary malignancies, EBUS-TBNA provides predominantly cytological specimens, which may be insufficient in conditions requiring preservation of tissue architecture and histopathological assessment [4]. The limited tissue volume and absence of intact nodal architecture may restrict definitive diagnosis in diseases such as lymphoma, sarcoidosis, tuberculosis, and other benign or uncommon mediastinal pathologies [5]. Various strategies have been proposed to overcome these limitations, including the use of different needle gauges, core biopsy needles, and intranodal forceps biopsy; however, the challenge of obtaining adequate histological material remains unresolved [6].

More recently, EBUS-guided transbronchial mediastinal cryobiopsy (EBUS-TBMC) has emerged as an innovative technique for obtaining larger and better-preserved tissue samples from mediastinal lymph nodes. In this procedure, following needle puncture or a small electrocautery-assisted incision, a cryoprobe is advanced into the target lymph node under real-time ultrasound guidance to obtain tissue samples. Compared with EBUS-TBNA, EBUS-TBMC yields larger specimens with preserved tissue architecture and fewer crush artifacts, thereby potentially improving diagnostic accuracy, particularly in diseases where histological evaluation is essential [4,5,7]. Consequently, EBUS-TBMC has gained increasing attention as a promising complementary or alternative technique for mediastinal sampling.

To date, comparative studies evaluating EBUS-TBNA and EBUS-TBMC have largely included heterogeneous patient populations, frequently encompassing individuals with primary lung masses in addition to mediastinal lymphadenopathy [2,4,8,9,10]. In patients presenting solely with mediastinal lymphadenopathy, distinguishing between malignant and benign etiologies poses a particular diagnostic challenge, especially in cases of granulomatous diseases and lymphoma where adequate histological architecture is crucial [11]. Although several studies have evaluated the diagnostic performance of EBUS-TBNA in patients with isolated mediastinal lymphadenopathy [12,13,14], data specifically assessing the role of EBUS-TBMC in this distinct population remain extremely limited. To the best of our knowledge, no prospective study has directly compared EBUS-TBNA and EBUS-TBMC exclusively in patients with isolated mediastinal lymphadenopathy without an accompanying lung parenchymal mass.

Therefore, our prospective cohort study aimed to directly compare the diagnostic yield, tissue adequacy, and complication profiles of EBUS-TBNA and EBUS-TBMC performed sequentially in the same session in patients with isolated mediastinal lymphadenopathy without an accompanying lung parenchymal mass. We hypothesized that, in this selected population, EBUS-TBMC would provide superior histological adequacy and diagnostic performance compared with EBUS-TBNA, without compromising procedural safety.

## 2. Materials and Method

### 2.1. Study Design and Setting

This prospective, single-center, observational cohort study was conducted at the Department of Pulmonology, Pamukkale University Hospital, between August 2025 and February 2026. The study included consecutive patients who underwent endobronchial ultrasound EBUS-TBNA followed by EBUS-TBMC for the evaluation of mediastinal lymphadenopathy.

The study protocol was approved by the Institutional Ethics Committee (Date: 26 August 2025, No: E-60116787-020-741690), and written informed consent was obtained from all participants prior to the procedure.

### 2.2. Patient Selection

Patients were eligible if they were ≥18 years of age, had mediastinal lymph nodes with a short-axis diameter ≥ 10 mm on thoracic computed tomography (CT), and provided written informed consent for bronchoscopic intervention. No preselection was made based on suspected malignant or benign etiology.

Patients with a detectable lung parenchymal mass were excluded from the study in order to establish a homogeneous cohort consisting exclusively of isolated mediastinal lymphadenopathy. No predefined nodal station was targeted. In each patient, the lymph node with the largest short-axis diameter was selected for sampling.

### 2.3. Bronchoscopic Procedure

All procedures were performed in the operating room under inpatient conditions with standard hospital admission, and patients were monitored continuously for cardiopulmonary parameters under moderate sedation. Airway management was achieved using flexible bronchoscopy under local anesthesia with conscious sedation: neither endotracheal intubation nor rigid bronchoscopy was routinely employed. Rapid on-site evaluation (ROSE) was not available due to technical limitations during the study period. EBUS-TBNA was performed first in all patients, followed by EBUS-TBMC in the same session. This standardized sequence was adopted based on previous studies suggesting that performing cryobiopsy prior to TBNA may negatively affect the diagnostic performance of subsequent needle aspiration due to procedure-related bleeding and tissue disruption [4].

**Figure 1:**
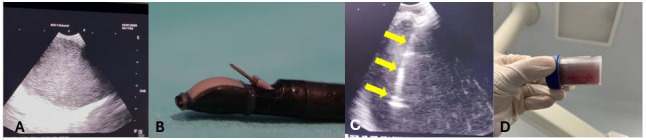
**Stepwise procedure of EBUS-TBNA sampling.** (**A**): Target lymph node station identified on endobronchial ultrasound imaging. (**B**) Aspiration needle attached to the EBUS scope. (**C**) Real-time ultrasound image showing advancement of the aspiration needle into the target lymph node during EBUS-TBNA; the needle trajectory is indicated by yellow arrows. (**D**) Obtained biopsy specimen placed in a tube, ready for diagnostic analysis.

EBUS-TBNA was performed first in all patients using a standard convex-probe endobronchial ultrasound bronchoscope. After real-time ultrasonographic identification and characterization of the target mediastinal lymph node, transbronchial needle aspiration was carried out using a dedicated 22-gauge EBUS-TBNA needle (SonoTip EBUS Pro, Mediglobe^®^, Achenmühle, Germany) (Figure 1A,B).

Following sonographic confirmation of needle position within the lymph node, four needle passes were systematically performed for each sampled lymph node station using standard aspiration technique (Figure 1C,D).

**Figure 2:**
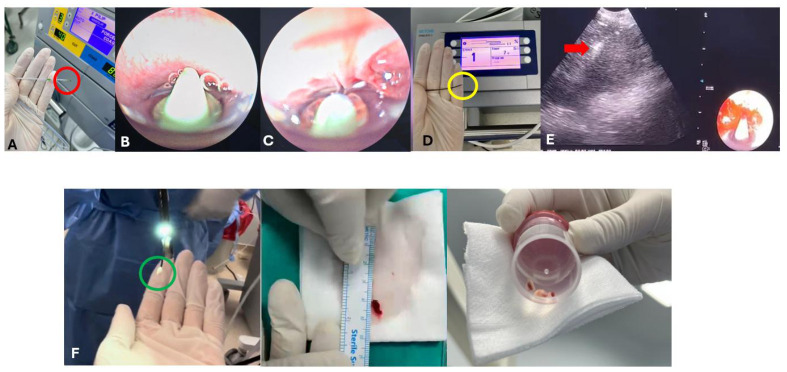
**Stepwise procedure of EBUS-TBMC sampling.** (**A**) Electrocautery needle knife, with the needle tip highlighted within the red circle. (**B**,**C**) Opening of the incision with the needle knife. (**D**) Cryoprobe (1.1 mm), with the probe tip indicated within the yellow circle. (**E**) Real-time bronchoscopic view during transbronchial mediastinal cryobiopsy; the biopsy site within the lymph node is indicated by a red arrow. (**F**) Collected biopsy specimens, demonstrating the frozen tissue immediately after extraction; the specimen is highlighted within a green circle.

EBUS-TBMC was subsequently performed following completion of EBUS-TBNA within the same procedural session. At the exact site of the prior TBNA puncture, a dedicated electrocautery needle knife (ENDO-FLEX GmbH, Voerde, Germany) was used to create a controlled incision through the bronchial wall under real-time EBUS guidance (Figure 2A–C). Subsequently, a 1.1-mm flexible cryoprobe (Erbecryo 20402-401, Erbe Elektromedizin, Tübingen, Germany) (Figure 2D) was introduced through the working channel of the EBUS bronchoscope and advanced through the incision tract created at the TBNA entry point into the target lymph node (Figure 2E). The procedure was carried out using Endo Cut I and forced coagulation modes, with Endo Cut effect setting +2, forced coagulation effect setting +3, and a maximum power output of 40 W. Correct positioning of the cryoprobe tip within the lymph node was confirmed by real-time ultrasonographic visualization. For each sampled lymph node station, three consecutive cryobiopsy specimens were obtained. The cryoprobe was activated for 7 s during each pass, after which the bronchoscope and cryoprobe were withdrawn en bloc to retrieve the specimen (Figure 2F). After specimen retrieval, the incision site was carefully inspected for bleeding or other immediate complications.

Bleeding during EBUS-TBNA and EBUS-TBMC procedures was prospectively recorded and classified according to the estimated volume of blood loss. Bleeding severity was categorized as mild (<10 mL), moderate (10–40 mL), or severe (>40 mL) [9]. The estimation of bleeding volume was based on the amount of blood aspirated through the bronchoscope suction channel and the volume collected in the suction canister during the procedure. All bleeding events were documented immediately following each sampling technique and evaluated separately for EBUS-TBNA and EBUS-TBMC.

All patients underwent routine post-procedural chest radiography to screen for procedure-related thoracic complications. In cases where radiographic findings were suspicious or equivocal, thoracic computed tomography (CT) was performed for confirmation. Pneumothorax and pneumomediastinum were defined as the presence of free air in the pleural or mediastinal space on CT imaging. Only CT-confirmed cases were recorded as complications for analysis.

### 2.4. Pathological Evaluation

Aspirated materials obtained by EBUS-TBNA were delivered to the pathology laboratory in tubes containing “Red solution”. Cytological preparations were generated using a Thermo Cytospin 4 device (Thermo Scientific, Waltham, MA, USA). The resulting smears were stained with Papanicolaou stain and evaluated for cytomorphological features. Cell blocks were prepared from the cellular sediment obtained after centrifugation, and 3–4 µm sections were cut and stained with hematoxylin and eosin (H&E).

Biopsy specimens obtained by EBUS-TBMC were fixed in 10% neutral buffered formalin, routinely processed, and embedded in paraffin. Sections (3–4 µm) were prepared and stained with H&E. Immunohistochemical and/or histochemical stains were performed when diagnostically indicated.

All cytological and histological specimens were evaluated without procedural identifiers in order to minimize interpretation bias. However, because of the inherent differences in specimen architecture and tissue volume between EBUS-TBNA and EBUS-TBMC samples, complete blinding to sampling modality could not be fully guaranteed (single-blind design).

**Figure 3:**
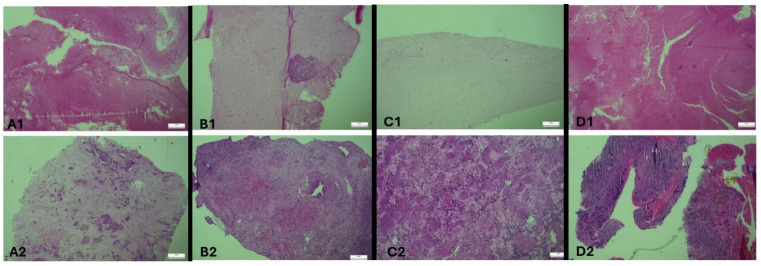
Comparison of Paired Histopathological Findings from EBUS-TBNA and EBUS-TBMC in Representative Patients. (**A1**) EBUS-TBNA specimen insufficient for diagnosis, showing only fibrin. H&E stain, original magnification ×50. (**A2**) EBUS-TBMC showing metastasis of a malignant epithelial tumor. (**B1**) EBUS-TBNA yielding a very limited tissue sample with only focal representation of the lesion. H&E stain, original magnification ×50. (**B2**) EBUS-TBMC specimen diagnostic of Hodgkin lymphoma. (**C1**) EBUS-TBNA specimen inadequate for diagnosis, showing fibrin. H&E stain, original magnification ×100. (**C2**) EBUS-TBMC demonstrating non-necrotizing granulomatous inflammation suggestive of sarcoidosis. (**D1**) EBUS-TBNA specimen insufficient for diagnosis, showing fibrin. H&E stain, original magnification ×50. (**D2**) EBUS-TBMC demonstrating reactive lymph node tissue.

Figure 3 presents representative histopathologic images from four different patients, comparing specimens obtained by EBUS-TBNA and EBUS-TBMC.

### 2.5. Outcome Measures

The primary outcome was diagnostic yield (proportion of cases in which a specific pathological diagnosis was established).

Secondary outcomes included tissue adequacy/diagnostic, complication rates (bleeding, pneumothorax, pneumomediastinum, etc.), and the need for additional diagnostic procedures. Given the substantial overlap between tissue adequacy and establishment of a definitive pathological diagnosis in our cohort, diagnostic yield was used as the principal indicator of sampling adequacy.

No third independent diagnostic gold standard was uniformly applied.

In cases where a specific diagnosis was established, the diagnostic process was considered complete. In cases without a definitive diagnosis, further evaluation (mediastinoscopy, surgical biopsy, or clinical–radiological follow-up) was performed based on PET-CT findings, multidisciplinary team decision, and patient consent. Final diagnosis was determined based on histopathological findings and/or clinical follow-up.

#### Statistical Analysis Section

Statistical analyses were conducted using IBM SPSS Statistics version 25.0 (IBM Corp., Armonk, NY, USA). Descriptive statistics for continuous variables were reported as mean ± standard deviation and minimum–maximum values, whereas categorical variables were summarized as frequencies (*n*) and percentages (%). Paired binary categorical variables were analyzed using the McNemar test. For paired categorical variables with an ordinal structure consisting of more than two levels, the McNemar–Bowker test was used to compare paired ordinal outcomes between EBUS procedures. The *p* value of <0.05 was considered statistically significant.

## 3. Results

**Figure 4:**
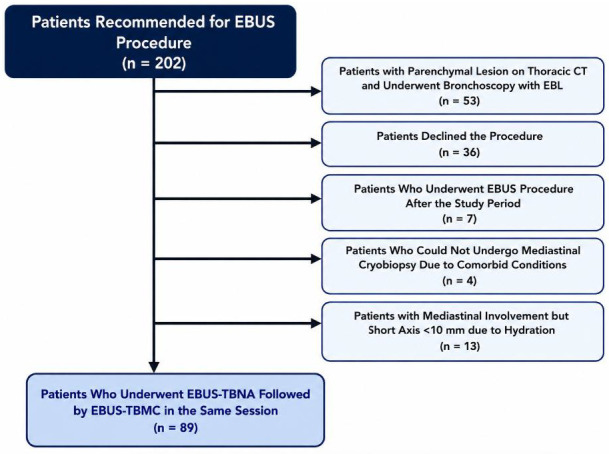
Flow diagram illustrating patient selection and study inclusion process.

A total of 89 patients were included in the study (Figure 4). Baseline demographic and clinical characteristics are summarized in Table 1. The cohort had a mean age of 57.8 ± 12.4 years and was nearly evenly distributed by sex. Approximately half of the patients were non-smokers, and the remaining patients were either current or ex-smokers. The most frequently sampled lymph node stations included 11L and station 7, followed by 4R, 11R, and 4L. The mean lymph node size was 21.2 ± 7.6 mm, and the mean TBMC specimen size was 4.2 ± 0.8 mm.

EBUS-TBMC demonstrated a markedly higher diagnostic yield compared with EBUS-TBNA. While EBUS-TBNA established a definitive diagnosis in 25 of 89 patients (28%), EBUS-TBMC achieved diagnostic adequacy in 74 patients (83.1%), corresponding to an absolute increase of 55.1%. Notably, EBUS-TBMC provided an additional diagnosis in 49 patients (55.1%) in whom EBUS-TBNA was non-diagnostic, whereas no cases were diagnosed exclusively by EBUS-TBNA. The difference in paired proportions was statistically significant (McNemar test, *p* < 0.001) (Table 2). In the EBUS-TBMC group, a definitive diagnosis was established in 71 of 82 patients (86.6%) when three cryobiopsy specimens were obtained, compared with 3 of 7 patients (42.8%) in whom fewer than three samples could be collected. Incomplete sampling was primarily due to procedure-related bleeding, while in one patient the procedure was terminated after two samples because of technical device malfunction.

When specific pathological diagnoses were analyzed, EBUS-TBMC demonstrated a broader diagnostic spectrum than EBUS-TBNA, particularly in granulomatous diseases, lymphoid malignancies, and benign reactive conditions. Notably, lymphoma and tuberculosis were diagnosed exclusively by TBMC. Detailed distributions of pathological diagnoses are presented in Table 3.

Bleeding complications were more frequent with EBUS-TBMC compared with EBUS-TBNA. While no bleeding was observed in 49.4% of TBNA procedures, this rate decreased to 34.8% with TBMC. Moderate and severe bleeding occurred more commonly following TBMC (21.3% and 10.1%, respectively) than TBNA (14.6% and 3.4%, respectively). The difference in paired bleeding severity distributions was statistically significant (McNemar test, *p* = 0.003) (Table 4). Importantly, in 80 patients no clinically significant bleeding was observed. In cases classified as mild bleeding, the duration was less than 1 min and resolved spontaneously without intervention. Bleeding exceeding 40 mL occurred in nine patients; in all of these cases, hemostasis was successfully achieved with cold saline instillation and topical adrenaline administration. No patient required surgical intervention, blood transfusion, escalation of care, or premature termination of the procedure. Although TBMC was associated with a higher incidence of bleeding events, all episodes were effectively managed endoscopically and no major hemorrhagic complications occurred.

One patient (1.1%) developed pneumomediastinum, confirmed by thoracic CT. No cases of pneumothorax were observed. The patient was managed conservatively with supplemental oxygen therapy and empirical third-generation cephalosporin treatment. No invasive intervention was required, and complete clinical and radiological resolution occurred during follow-up.

Additional diagnostic procedures were required in 8 of 15 patients with non-diagnostic initial sampling results. Mediastinoscopy was performed in three patients, yielding diagnoses of small-cell lung carcinoma, B-cell lymphoma, and sinus histiocytosis. One patient underwent repeat bronchoscopy with bronchial mucosal biopsy, which established a diagnosis of tuberculosis. Two patients underwent chest wall core-needle biopsy following the development of pleural abnormalities during follow-up, resulting in diagnoses of mesothelioma and squamous cell carcinoma. Video-assisted thoracoscopic surgery (VATS) was performed in two patients, revealing breast cancer metastasis and non-small-cell lung carcinoma, respectively.

Patients diagnosed with reactive lymph node hyperplasia or anthracotic pigment deposition were not considered definitively benign solely based on pathological findings. These patients underwent clinical and radiological follow-up according to routine institutional practice. In patients who had completed follow-up evaluation, control thoracic CT examinations performed approximately 3–6 months after the procedure demonstrated no significant increase in mediastinal lymph node size, newly developed pathological findings, or clinical/radiological progression suggestive of malignancy. Therefore, additional invasive diagnostic procedures such as mediastinoscopy were not considered necessary during the current follow-up period. However, several patients have not yet completed long-term follow-up, and clinical surveillance is ongoing.

Among the remaining patients, one declined further invasive diagnostic procedures despite persistent radiological suspicion of malignancy, three demonstrated stable lymph node size during radiological follow-up, and two were lost to follow-up.

## 4. Discussion

In this prospective head-to-head cohort study of patients with isolated mediastinal lymphadenopathy, EBUS-TBMC demonstrated a markedly higher diagnostic yield than EBUS-TBNA (83.1% vs. 28%). TBMC established a definitive diagnosis in 74 of 89 patients, whereas TBNA was diagnostic in only 25 cases. Importantly, TBMC provided an additional diagnosis in 49 patients in whom TBNA was non-diagnostic, and no patient was diagnosed exclusively by TBNA. The difference between the two techniques was statistically significant (*p* < 0.001), underscoring the substantial incremental diagnostic contribution of cryobiopsy in this clinical setting. Beyond the quantitative difference in diagnostic yield, the fundamental advantage of EBUS-TBMC lies in its ability to obtain larger, architecturally preserved tissue samples. This is particularly critical in diseases such as lymphoma and sarcoidosis, where histopathological diagnosis relies not only on cytology but also on the evaluation of tissue architecture, granuloma formation, and cellular distribution patterns, features that are frequently insufficiently captured by needle aspiration techniques. In this context, the limited tissue volume and loss of histological architecture inherent to EBUS-TBNA represent a major diagnostic constraint, particularly in non-malignant and lymphoproliferative disorders.

The superiority of cryobiopsy is not merely incremental but reflects a qualitative difference in the type of tissue obtained, shifting the diagnostic approach from cytology-based assessment to true histopathological evaluation. Our findings are consistent with the growing body of evidence demonstrating the superior diagnostic performance of mediastinal cryobiopsy compared with conventional needle aspiration. Previous studies have similarly reported significantly higher diagnostic yields with EBUS-guided cryobiopsy. In a large cohort, cryobiopsy achieved an overall diagnostic yield of 91.8% compared with 79.9% for TBNA (*p* = 0.001), with superiority in rare tumors and benign conditions, whereas no significant difference was observed in patients with advanced lung cancer [4]. Likewise, Mangold et al. reported a diagnostic yield of 91.2% for cryobiopsy versus 56.2% for TBNA (*p* < 0.001), highlighting its incremental value especially in benign diseases, rare tumors, and metastatic malignancies [2]. Randomized data from Fan and Zhang further demonstrated that combining EBUS-TBMC with TBNA significantly increased overall diagnostic yield compared to TBNA alone (93% vs. 81%, *p* = 0.0039) [15]. Moreover, in cases with inadequate ROSE assessment, cryobiopsy provided additional diagnostic contribution in more than half of patients [8]. A recent meta-analysis including 538 patients confirmed these findings, reporting higher diagnostic accuracy for TBMC compared with TBNA (89.6% vs. 77.1%), with particularly pronounced superiority in lymphoma (86.3% vs. 27.3%, *p* = 0.0006) and benign diseases (87.6% vs. 60.0%, *p* < 0.0001) [5]. These findings are further supported by the most recent meta-analysis by Kamath et al., which included 857 patients across eleven studies published up to 2025. In this comprehensive review, the overall diagnostic yield of EBUS-guided cryobiopsy was 91.9%, compared with 76.6% for EBUS-TBNA alone, without a significant increase in major complications. The authors also emphasized the advantage of larger tissue samples obtained with cryobiopsy, particularly for next-generation sequencing in lung cancer and for improving diagnostic accuracy in benign diseases and lymphoma [16].

Importantly, most prior studies included heterogeneous populations with concomitant pulmonary mass lesions or advanced lung cancer, in whom TBNA already performs well. In contrast, our study exclusively enrolled patients with isolated mediastinal lymphadenopathy without parenchymal masses, representing a diagnostically more challenging subgroup. In this specific population, the incremental value of TBMC was even more pronounced, while TBNA alone demonstrated limited diagnostic yield. This distinction is particularly relevant in mass-negative mediastinal lymphadenopathy, where the differential diagnosis is often dominated by benign granulomatous diseases and lymphomas, entities in which adequate tissue architecture is essential for definitive diagnosis and subclassification. Therefore, rather than replacing TBNA universally, our findings suggest that TBMC may serve as a complementary or early adjunctive strategy, particularly in patients with suspected benign disease, lymphoma, or mass-negative mediastinal lymphadenopathy. This approach may also reduce the need for more invasive diagnostic procedures such as mediastinoscopy or VATS in selected patients. Nevertheless, a limited number of patients in our cohort still required additional invasive diagnostic procedures, including mediastinoscopy or VATS, following non-diagnostic endobronchial sampling. These findings suggest that although TBMC may substantially reduce the need for surgical diagnostic interventions, invasive procedures may still be necessary in selected unresolved cases.

The relatively low diagnostic yield of EBUS-TBNA observed in our cohort (28%) also warrants careful consideration. This rate is lower than that reported in many conventional EBUS-TBNA series, including studies focused on isolated mediastinal lymphadenopathy and sarcoidosis, in which diagnostic yields frequently exceed 70–80% under optimized sampling conditions [17,18]. Several factors may explain this finding. First, our study exclusively included patients with isolated mediastinal lymphadenopathy without accompanying parenchymal lung masses, a subgroup enriched for benign granulomatous diseases and lymphoproliferative disorders in which cytology-based techniques are known to have lower sensitivity and often require preserved tissue architecture for definitive diagnosis. Second, sampling was intentionally restricted to a single target lymph node per patient in order to enable direct paired comparison between EBUS-TBNA and EBUS-TBMC under identical pathological conditions. While this design minimized inter-nodal heterogeneity, it may have reduced the cumulative diagnostic yield typically achieved through multi-station sampling in routine clinical practice. In addition, rapid on-site cytological evaluation (ROSE) was not available because of institutional logistical limitations, which may also have contributed to lower TBNA adequacy rates. Finally, cytopathological evaluation was performed in routine clinical practice conditions. Although all specimens were reviewed by a pathologist routinely involved in thoracic cytopathology, the interpretation of limited cytological material particularly in benign granulomatous and lymphoid disorders remains highly expertise-dependent and may vary according to the level of subspecialized cytopathology experience. Taken together, these factors likely contributed to the lower diagnostic performance of TBNA in our study and should be considered when interpreting the magnitude of difference observed between the two techniques.

Not all studies, however, have demonstrated a statistically significant advantage of cryobiopsy over TBNA. In a prospective study by Madan et al., EBUS-guided mediastinal cryobiopsy and intranodal forceps biopsy were evaluated as adjunctive techniques to TBNA in 34 patients. Although both additional sampling methods showed numerically higher specimen adequacy and diagnostic yield compared with TBNA, the differences did not reach statistical significance (*p* = 0.56 and *p* = 0.38, respectively). No major complications were reported. The authors concluded that the incremental diagnostic contribution of these more invasive techniques might be limited [19]. However, the relatively small sample size likely reduced the statistical power of the study, limiting the generalizability of these findings. In contrast, our cohort demonstrated a robust and statistically significant improvement in diagnostic yield with TBMC, suggesting that patient selection and study design may critically influence observed outcomes.

From a technical standpoint, several approaches to EBUS-guided mediastinal cryobiopsy have been described. The first reported case by Zhang et al. [20] in 2020, performed in a 17-year-old patient with mediastinal seminoma, utilized a high-frequency electrocautery needle knife to create an entry tract for the cryoprobe. Following this initial description, two principal technical strategies emerged. One group of operators continued to employ electrocautery-assisted incision to facilitate cryoprobe access [4,8,15,21], while another adopted a less invasive approach by advancing the cryoprobe directly through the pre-existing TBNA needle tract after standard EBUS-TBNA puncture [7,9,22,23]. The latter technique has become the more widely implemented method in clinical practice due to its procedural simplicity. In our study, we followed the electrocautery-assisted approach, creating a controlled incision at the TBNA puncture site prior to cryoprobe insertion, aiming to ensure stable and reproducible access to the target lymph node.

An important methodological consideration of our study is that EBUS-TBNA and EBUS-TBMC were performed sequentially during the same procedural session and within the same target lymph node. This design may introduce potential sources of bias. Prior TBNA sampling could theoretically cause partial tissue depletion or procedure-related bleeding that may influence subsequent cryobiopsy performance, while the initial needle tract may also facilitate cryoprobe access to the lymph node. Conversely, performing the procedures in separate lymph nodes or different patient cohorts would introduce substantially greater heterogeneity related to nodal architecture, disease distribution, and tissue composition. Therefore, sequential sampling of the same lymph node was intentionally preferred to provide the most direct head-to-head comparison between the two techniques under identical pathological conditions. Importantly, this methodological approach is consistent with the currently published EBUS-TBMC literature, in which TBNA is routinely performed before cryobiopsy to establish the access tract and guide cryoprobe insertion [2,8,9,23]. Nevertheless, the possibility that sequential sampling may have influenced both diagnostic yield and complication profiles particularly bleeding-related outcomes should be acknowledged when interpreting our findings.

Although the literature on EBUS-TBMC has primarily focused on specimen size and architectural preservation as key determinants of diagnostic success, variability in the number of cryobiopsy passes across studies suggests that sampling quantity may also influence diagnostic yield. Reported series include protocols ranging from a single cryobiopsy [15] specimen to as many as four passes per lymph node [8,24]. A direct head-to-head comparison of diagnostic success according to sample number across studies could theoretically clarify the independent contribution of cumulative sampling. However, such comparisons are methodologically challenging because centers differ substantially with respect to freezing time, cryoprobe diameter (e.g., 1.1-mm vs. 1.7-mm), access technique, and patient selection, all of which independently affect specimen size and quality [25].

Within our cohort, a definitive diagnosis was established in 71 of 82 patients (86.6%) in whom three cryobiopsy specimens were obtained, whereas only 3 of 7 patients (42.8%) in whom fewer than three samples could be collected achieved diagnostic confirmation. Although the small number of patients in the reduced-sample group precludes definitive conclusions, the numerical difference suggests that cumulative sampling may contribute to diagnostic adequacy in addition to specimen size alone.

This observation is consistent with emerging evidence indicating that procedural variables, including the number of cryobiopsy passes, may influence diagnostic performance. Recent studies evaluating EBUS-TBMC have demonstrated a positive association between the number of cryo-passes and diagnostic yield, with diagnostic success increasing substantially when two or more samples are obtained. In an exploratory analysis of TBMC procedures, diagnostic yield increased from 33% with a single pass to approximately 85% when two to three cryobiopsies were performed, suggesting that cumulative tissue acquisition may play a critical role in achieving diagnostic adequacy [26]. Given the modest sample size of our study, this observation should be interpreted cautiously. Nevertheless, it raises the hypothesis that, beyond tissue dimensions and freezing parameters, the planned number of cryobiopsy passes may represent an independent procedural variable worthy of systematic evaluation in future prospective, standardized studies.

Regarding procedural safety, EBUS-TBMC demonstrates a complication profile comparable to other EBUS-based techniques and remains substantially less invasive than surgical approaches such as mediastinoscopy or VATS [27].

Another important point is the management of patients with benign pathological findings such as reactive lymph node hyperplasia or anthracotic pigment deposition. In our cohort, these findings were not accepted as definitively benign solely based on histopathological evaluation. Instead, patients underwent clinical and radiological follow-up according to institutional practice. Follow-up thoracic CT imaging did not demonstrate radiological progression, newly developed suspicious findings, or clinical deterioration suggestive of occult malignancy in patients who completed surveillance. Therefore, additional invasive procedures such as mediastinoscopy were not routinely required during the current follow-up period. Nevertheless, continued long-term follow-up remains important, particularly in patients with incomplete surveillance data or persistent clinical suspicion.

In the most recent meta-analysis of EBUS-TBMC, mild bleeding was the most common complication (36.5%), typically resolving spontaneously without the need for bronchoscopic intervention, while clinically significant bleeding requiring hemostatic measures was rare (0.7%) [28].

In our cohort, bleeding events were observed more frequently following TBMC than TBNA, including severe bleeding episodes in approximately 10% of patients according to predefined volumetric criteria. Although all bleeding events were successfully controlled bronchoscopically using topical cold saline and adrenaline without the need for transfusion, surgical intervention, or escalation of care, these findings should not be interpreted as clinically negligible. The relatively higher bleeding burden associated with TBMC likely reflects the larger tissue samples obtained and the additional airway wall incision required for cryoprobe access. Importantly, because EBUS-TBNA and EBUS-TBMC were performed sequentially within the same procedural session, precise attribution of bleeding events to a single technique remains inherently challenging [27]. Prior TBNA puncture may itself contribute to mucosal disruption or facilitate subsequent bleeding during cryobiopsy. Therefore, the true independent bleeding risk attributable specifically to TBMC cannot be fully determined from the present study design and should be interpreted cautiously.

Nevertheless, despite the increased bleeding frequency, no life-threatening hemorrhage, intensive care requirement, or procedure-related mortality occurred in our cohort. These findings suggest that TBMC can be performed with acceptable safety in experienced centers, although careful patient selection and procedural expertise remain essential.

Taken together, our findings support the concept that cryobiopsy does not merely increase tissue quantity but may fundamentally improve tissue architecture preservation, allowing more accurate histopathological subtyping and ancillary testing. Rather than positioning TBMC as a universal replacement for TBNA, our data support a complementary or stepwise diagnostic approach, particularly in patients with suspected lymphoma, granulomatous disease, or isolated mediastinal lymphadenopathy in whom cytology alone may be insufficient for definitive diagnosis.

One of the principal strengths of our study is the highly selected and clinically challenging patient population. Unlike most previous studies that included heterogeneous cohorts with concomitant pulmonary masses or advanced lung cancer, our cohort consisted exclusively of patients with isolated mediastinal lymphadenopathy without parenchymal lung lesions. This focused design minimized disease heterogeneity and allowed direct evaluation of EBUS-TBMC in a subgroup where histological tissue architecture is particularly critical for diagnosis. In addition, the paired same-node comparison design provided a controlled head-to-head assessment of both techniques under identical pathological conditions.

Several limitations should be acknowledged. First, this was a single-center study, which may limit the generalizability of our findings. However, our institution represents a high-volume tertiary referral center with substantial experience in advanced interventional pulmonology techniques and is among the few centers in the region routinely performing EBUS-TBMC. Therefore, the relatively large cohort presented here provides valuable real-world data from a specialized setting where procedural expertise is well established.

Another limitation relates to the pathological evaluation process. Although specimens were submitted without explicit indication of the sampling technique, the inherent differences in specimen size and tissue architecture between cytological and cryobiopsy samples may have partially compromised blinding by allowing indirect recognition of the sampling modality during pathological assessment. In addition, rapid on-site cytological evaluation (ROSE) was not available because of institutional logistical limitations, which may have negatively influenced the diagnostic adequacy of EBUS-TBNA.

## Figures and Tables

**Table 1:** Demographic and Clinical Characteristics of the Study Population.

		*n*	% (Percentage)
Sex	Female	43	48.3
	Male	46	51.7
Age, year	Mean (±S.D)		57.79 ± 12.44
Smoking History	Non-smoker	42	47.2
	Ex-smoker	26	29.2
	Smoker	21	23.6
Lymph node station	4R	20	22.5
	4L	2	2.2
	7	26	29.2
	11R	8	9
	11L	30	33.7
			**mm**
Lymph node size	Mean (±S.D)		21.18 ± 7.61
	Minimum-Maximum		9.62–46.1
TBMC size	Mean (±S.D)		4.19 ± 0.79
	Min–Max		3–6

**Table 2:** Diagnostic Yield of EBUS-TBNA and EBUS-TBMC.

		EBUS_MKB		*p*
		Non-Diagnostic/Preliminary Diagnosis (*n* = 15)	Diagnostic (*n* = 74)	
EBUS_TBNA	Non-diagnostic/preliminary diagnosis (*n* = 64)	15 (16.9%)	49 (55.1%)	0.0001 *
	Diagnostic (*n* = 25)	0 (0%)	25 (28.1%)	

* *p* < 0.05 statistically significant; *p*-value was calculated using the McNemar test for paired binary categorical data.

**Table 3:** Distribution of pathological diagnoses obtained by EBUS-TBNA and EBUS-TBMC.

Diagnosis	EBUS-TBNA *n*/(%)	EBUS-TBMC *n*/(%)
Sarcoidosis	7 (7.9)	28 (32.5)
Metastatic malignancy	3 (3.4)	11 (12.4)
Primary lung cancer (adenocarcinoma/NSCLC/neuroendocrine)	0 (0)	5 (5.6)
Lymphoma	0 (0)	5 (5.6)
Tuberculosis	0 (0)	2 (2.2)
Foreign body–type granuloma	0 (0)	1 (1.1)
Sinus histiocytosis	0 (0)	2 (2.2)
Anthracotic pigment	2 (2.2)	3 (3.4)
Malignancy negative/reactive lymph node	13 (14.6)	17 (19.1)
Non-diagnostic/insufficient sampling	64 (71.9)	15 (16.9)
Total	**89 (100)**	**89 (100)**

**Table 4:** Procedure-related complications and bleeding severity following EBUS-TBNA and EBUS-TBMC.

		EBUS-TBMC Bleeding				
		None (*n* = 31)	Mild (<10 mL) (*n* = 30)	Moderate (10–40 mL) (*n* = 19)	Severe (>40 mL) (*n* = 9)	*p*
EBUS-TBNA bleeding	None (*n* = 44)	28 (31.5%)	13 (14.6%)	3 (3.4%)	0 (0%)	0.003 *
	Mild (<10 mL) (*n* = 29)	3 (3.4%)	13 (14.6%)	10 (11.2%)	3 (3.4%)	
	Moderate (10–40 mL) (*n* = 13)	0 (0%)	4 (4.5%)	6 (6.7%)	3 (3.4%)	
	Severe (>40 mL) (*n* = 3)	0 (0%)	0 (0%)	0 (0%)	3 (3.4%)	

* *p* < 0.05 statistically significant; *p*-value for bleeding severity was calculated using the McNemar–Bowker test for paired ordinal categorical data.

## Data Availability

The original contributions presented in this study are included in the article. Further inquiries can be directed to the corresponding author.

## References

[1] De Leyn P., Dooms C., Kuzdzal J., Lardinois D., Passlick B., Rami-Porta R., Turna A., Schil P.V., Venuta F., Waller D. (2014). Revised ESTS guidelines for preoperative mediastinal lymph node staging for non-small-cell lung cancer. Eur. J. Cardio-Thorac. Surg..

[2] Mangold M.S., Franzen D.P., Hetzel J., Latshang T.D., Roeder M., Vesenbeckh S.M., Ulrich S., Gaisl T., Steinack C. (2024). Ultrasound-guided transbronchial cryobiopsy of mediastinal and hilar lesions: A multicenter pragmatic cohort study with real-world evidence. BMJ Open Respir. Res..

[3] Ugurlu E., Metin M., Cetin N., Kilicarslan E., Degirmencioglu S., Sengoz T., Akbudak I.H., Dogu G.G., Aydogmus U. (2023). Evaluation of hypermetabolic mediastinal-hilar lymph nodes determined by PET/CT with EBUS-TBNA and calculation of SUVmax cutoff values in differentiation of malignancy. Medicine.

[4] Zhang J., Guo J.-R., Huang Z.-S., Fu W.-L., Wu X.-L., Wu N., Kuebler W.M., Herth F.J., Fan Y. (2021). Transbronchial mediastinal cryobiopsy in the diagnosis of mediastinal lesions: A randomised trial. Eur. Respir. J..

[5] Zhang Z., Li S., Bao Y. (2024). Endobronchial Ultrasound-Guided Transbronchial Mediastinal Cryobiopsy versus Endobronchial Ultrasound-Guided Transbronchial Needle Aspiration for Mediastinal Disorders: A Meta-Analysis. Respiration.

[6] Ramarmuty H.Y., Oki M. (2024). Endobronchial ultrasound-guided transbronchial mediastinal cryobiopsy: A narrative review. Mediastinum.

[7] Gonuguntla H.K., Shah M., Gupta N., Agrawal S., Poletti V., Nacheli G.C. (2021). Endobronchial ultrasound-guided transbronchial cryo-nodal biopsy: A novel approach for mediastinal lymph node sampling. Respirol. Case Rep..

[8] Maturu V.N., Prasad V.P., Vaddepally C.R., Dommata R.R., Sethi S. (2024). Endobronchial Ultrasound-guided Mediastinal Lymph Nodal Cryobiopsy in Patients with Nondiagnostic/Inadequate Rapid On-site Evaluation. J. Bronchol. Interv. Pulmonol..

[9] Ariza-Prota M., Pérez-Pallarés J., Fernández-Fernández A., García-Alfonso L., Cascón J.A., Torres-Rivas H., Fernández-Fernández L., Sánchez I., Gil M., García-Clemente M. (2023). Endobronchial ultrasound-guided transbronchial mediastinal cryobiopsy in the diagnosis of mediastinal lesions: Safety, feasibility and diagnostic yield—Experience in 50 cases. ERJ Open Res..

[10] Poletti V., Ravaglia C., Gurioli C., Piciucchi S., Dubini A., Cavazza A., Chilosi M., Rossi A., Tomassetti S. (2016). Invasive diagnostic techniques in idiopathic interstitial pneumonias. Respirology.

[11] Schwalk A.J., Grosu H. (2025). Approach to Isolated Mediastinal Lymphadenopathy. Clin. Chest Med..

[12] Temiz D., İn E., Kuluöztürk M., Kırkıl G., Artaş G., Turgut T., Deveci F. (2021). The role of endobronchial ultrasound-guided transbronchial needle aspiration in the differential diagnosis of isolated mediastinal and/or hilar lymphadenopathy. Diagn. Cytopathol..

[13] Evison M., Crosbie P.A.J., Morris J., Martin J., Barber P.V., Booton R. (2014). A study of patients with isolated mediastinal and hilar lymphadenopathy undergoing EBUS-TBNA. BMJ Open Respir. Res..

[14] Velu P.P., Reid P.A., Wallace W.A., Skwarski K.M. (2017). Isolated mediastinal lymphadenopathy—Performance of EBUS-TBNA in clinical practice. J. R. Coll. Physicians Edinb..

[15] Fan Y., Zhang A.-M., Wu X.-L., Huang Z.-S., Kontogianni K., Sun K., Fu W.-L., Wu N., Kuebler W.M., Herth F.J.F. (2023). Transbronchial needle aspiration combined with cryobiopsy in the diagnosis of mediastinal diseases: A multicentre, open-label, randomised trial. Lancet Respir. Med..

[16] Kamath S., Jahangir A., Daouk S., Youness H.A. (2025). Mediastinal lymph node cryobiopsy guided by endobronchial ultrasound: A comprehensive review of methods and outcomes. Mediastinum.

[17] Deng M., Zheng Z., Zhang X., Xia Y., Tang F., Yang Z., Zhong C., Tong R., Zhou G., Li X. (2026). EBUS-guided transbronchial mediastinal cryobiopsy for diagnosing non-metastatic lymphadenopathy: A randomized controlled trial. Med..

[18] Tyan C.C., Machuca T., Czarnecka K., Ko H.M., da Cunha Santos G., Boerner S.L., Pierre A., Cypel M., Waddell T., Darling G. (2017). Performance of Endobronchial Ultrasound-Guided Transbronchial Needle Aspiration for the Diagnosis of Isolated Mediastinal and Hilar Lymphadenopathy. Respiration.

[19] Madan M., Mahendran A., Kumar R., Kedia Y., Kaushik R., Ish P., Chakrabarti S., Gupta N.K., Gupta N. (2024). Comparative yield of transbronchial cryo-nodal biopsy, transbronchial intra-nodal forceps biopsy, and transbronchial needle aspiration for mediastinal lesions at a tertiary care center in India (COLD-FORCEPS study). Monaldi Arch. Chest Dis..

[20] Zhang J., Fu W.-L., Huang Z.-S., Guo J.-R., Li Q., Herth F.J., Fan Y. (2020). Primary Mediastinal Seminoma Achieved by Transbronchial Mediastinal Cryobiopsy. Respiration.

[21] Cheng T.-L., Huang Z.-S., Zhang J., Wang J., Zhao J., Kontogianni K., Fu W.-L., Wu N., Kuebler W., Herth F. (2024). Comparison of cryobiopsy and forceps biopsy for the diagnosis of mediastinal lesions: A randomised clinical trial. Pulmonology.

[22] Ramarmuty H.Y., Huan N.-C., Nyanti L.E., Khoo T.S., Renganathan T., Manoh A.Z., Azman N., Kannan K.K.S. (2024). Early experience of endobronchial ultrasound-guided transbronchial nodal cryobiopsy: A case series from Sabah, Malaysia. Ther. Adv. Respir. Dis..

[23] Poletti V., Petrarulo S., Piciucchi S., Dubini A., De Grauw A., Sultani F., Martinello S., Gonunguntla H., Ravaglia C. (2024). EBUS-guided cryobiopsy in the diagnosis of thoracic disorders. Pulmonology.

[24] Lobera E.S., Codeso F.P., Miranda E.C. (2023). Endobronchial ultrasound-guided transbronchial mediastinal cryobiopsy: Series of 50 cases. Rev. Clínica Española (Engl. Ed.).

[25] Gershman E., Amram Ikan A., Pertzov B., Rosengarten D., Kramer M.R. (2022). Mediastinal “deep freeze”—transcarinal lymph node cryobiopsy. Thorac. Cancer.

[26] Kho S.S., Tan S.H., Soo C.I., Ramarmuty H.Y.D., Chai C.S., Huan N.C., Ng K.L., Matsumoto Y., Poletti V., Tie S.T. (2024). An explorative analysis on the optimal cryo-passes and freezing time of the ultrathin cryoprobe in endobronchial ultrasound-guided transbronchial mediastinal cryobiopsy. Sci. Rep..

[27] Lentz R.J., Argento A.C., Colby T.V., Rickman O.B., Maldonado F. (2017). Transbronchial cryobiopsy for diffuse parenchymal lung disease: A state-of-the-art review of procedural techniques, current evidence, and future challenges. J. Thorac. Dis..

[28] Mathew R., Roy W.E., Thomas E.S., Meena N., Danilevskaya O. (2024). Meta-analysis and systematic review of mediastinal cryobiopsy versus endobronchial ultrasound-transbronchial needle aspiration (EBUS-TBNA) in the diagnosis of intrathoracic adenopathy. J. Thorac. Dis..

